# Determinants of experience & satisfaction in telehealth psychiatry during the COVID-19 pandemic for patients & providers

**DOI:** 10.3389/fpsyt.2023.1237249

**Published:** 2023-09-01

**Authors:** Michael Morreale, Ilana Cohen, Michael Van Wert, Alexis Beccera, Leslie Miller, William Narrow, Barbara Schweizer, Jason Straub, Peter Zandi, Anne Ruble

**Affiliations:** ^1^Mood Disorders Center, Department of Psychiatry and Behavioral Sciences, Johns Hopkins School of Medicine, Baltimore, MD, United States; ^2^Johns Hopkins Resident Outpatient Continuity Clinic, Department of Psychiatry and Behavioral Sciences, Johns Hopkins School of Medicine, Baltimore, MD, United States; ^3^Johns Hopkins Bayview Community Psychiatry Program, Department of Psychiatry and Behavioral Sciences, Johns Hopkins School of Medicine, Baltimore, MD, United States; ^4^Department of Psychiatry and Behavioral Sciences, Johns Hopkins Bayview Community Psychiatry Program, Johns Hopkins School of Medicine, Baltimore, MD, United States

**Keywords:** telepsychiatry, telemedicine, telehealth, quality improvement, survey

## Abstract

**Introduction:**

The objective of this study was to characterize the experiences and overall satisfaction of patients and providers with the March 2020 transition to telehealth in a psychiatric setting (telepsychiatry). The study also investigated how socio-demographic and clinical characteristics impact an individual’s experiences and satisfaction with telepsychiatry.

**Methods:**

Responses were collected from 604 patients and 154 providers engaged in clinical care at one of three participating Johns Hopkins Medicine outpatient psychiatric clinics between January 2020–March 2021. Survey data were collected by self-report via Qualtrics or telephone follow-up.

**Results:**

Respondents were predominately female and White. Over 70% of patients and providers were generally satisfied with telepsychiatry. However, providers were more likely to favor in-person care over telepsychiatry for post-pandemic care 48% to 17% respectively, while 35% rated both modalities equivalently. Patients were more evenly divided with 45% preferring telepsychiatry compared to 42% for in-person care, and only 13% rating them equivalently. Among providers, technical difficulties were significantly associated with both less satisfaction and lower preference for telepsychiatry [odds ratio for satisfaction (OR_S_) = 0.12; odds ratio for preference (OR_P_) = 0.13]. For patients, factors significantly associated with both lower satisfaction and lower preference for telepsychiatry included technical difficulties (OR_S_ = 0.20; OR_P_ = 0.41), unstable access to the internet (OR_S_ = 0.46; OR_P_ = 0.50), worsening depression (OR_S_ = 0.38; OR_P_ = 0.36), and worsening anxiety (OR_S_ = 0.41; OR_P_ = 0.40). Factors associated with greater satisfaction and higher preference for telepsychiatry among patients included higher education (OR_S_ = 2.13; OR_P_ = 1.96) and a decrease in technical difficulties over time (OR_S_ = 2.86; OR_P_ = 2.35).

**Discussion:**

Patients and providers were satisfied with telepsychiatry. However, there were greater differences between them in preferences for continuing to use telepsychiatry post-pandemic. These findings highlight factors that influence patient and provider preferences and should be addressed to optimize the use of telepsychiatry in the future.

## Introduction

The SARS-CoV-2 (COVID-19) pandemic caused unprecedented changes to healthcare services, including the rapid transition to telemedicine. Telemedicine refers to the provision of healthcare from a distance through information and communications technology (1). Telepsychiatry is the application of telehealth to psychiatric practice, encompassing services such as evaluations, individual or group therapy, psychoeducation, and medication management for behavioral health (2). The use of telepsychiatry and telemedicine in general was limited before the pandemic. For example, a study of over 200,000 privately insured and Medicare Advantage enrollees found that the overall rate of telemedicine encounters was quite low, reaching less than 7 per 1,000 enrollees in 2017 (3). Barriers to employing telepsychiatry prior to the pandemic included concerns about exacerbating challenges in access to care in some populations due to the digital divide, financial constraints, and difficulties faced by providers in understanding and adhering to varying state regulations (4–6).

Despite these concerns, telepsychiatry can have many advantages if implemented in a safe, effective manner that is acceptable to users and adaptable to specific facets of care across different specialties and clinics (2, 4, 7, 8). It is therefore crucial to identify what factors (e.g., dedicated technical support and additional clinical training) are likely to promote a telepsychiatry workflow that is user-friendly while still maintaining care efficacy and safety standards (2, 7, 8). In understanding these factors, both in the literature at large and within a specific clinic, healthcare systems can utilize telepsychiatry in a manner that promotes access to care and increased clinical efficiency in a sustainable and equitable manner (2, 4).

Research prior to the pandemic found that telemedicine consistently demonstrated treatment efficacy and patient acceptability that was comparable to in-person care. For instance, an evaluation of a telemedicine pilot program across five specialties, including psychiatry, found that the majority of patients (63%) and clinicians (59%) did not differentiate between virtual and office visits in terms of the overall quality of the visit (9). Subsequent studies continued to report overall high approval of telemedicine by both patients and providers, with Hubley et al. (10) noting telemedicine’s advantages of convenience and cost while maintaining comparable results to in-person appointments (3, 10–13).

During the pandemic, telepsychiatry utilization dramatically increased, accounting for almost 40% of all mental health and substance use outpatient visits at its peak (14). In contrast to pre-pandemic efforts, pandemic-era telepsychiatry was adopted rapidly and often with minimal planning or training, risking implementation of telepsychiatry in a manner that was neither sustainable nor on par with in-person care (7, 15). However, evaluations of telepsychiatry during this period continued to report high levels of satisfaction among patients and providers (6, 9, 16–21). Both groups identified many benefits, including ease of transportation and scheduling, lowered infection risk, and fewer cancellations (16, 17). Further, some providers noted increased feelings of safety while evaluating patients who may be prone to violence (20). However, ongoing challenges have persisted. For example, while using telehealth, providers must navigate interruptions in clinical care due to technological issues or distractions from the patient’s life (20, 22). Providers also report more challenges in establishing a therapeutic alliance and obtaining a holistic understanding of client health while using telehealth (20). Patient challenges predominantly include limited access to private spaces and technological issues (18). Despite the existing work dedicated to examining factors associated with successful telepsychiatry implementation, data are limited with regard to how patient and provider characteristics and clinical experiences help shape attitudes towards telepsychiatry. A greater understanding of these relationships will be crucial in determining how telepsychiatry has been successfully and equitably implemented during the pandemic, and where additional resources may be needed to help to optimize care.

The goal of the present study was to examine how the rapid transition to telepsychiatry impacted clinical care within a department of psychiatry from the perspective of both the patient and the provider. Specifically, we sought to examine the experiences of patients and providers with telepsychiatry during the COVID-19 pandemic and their preferences for telepsychiatry compared with in-person care for different aspects of treatment. Our work addresses previous limitations by investigating in greater detail patient and provider characteristics that are associated with their experiences and preferences for telepsychiatry. The ultimate goal of this work is to provide insights that can guide the practice of telepsychiatry by clarifying for whom it works well, what challenges persist, and if changes can be made to improve the quality of care delivered through telepsychiatry. These are urgent questions because the use of telepsychiatry has continued to grow beyond the public health crisis spurred by the COVID-19 pandemic.

## Methods

### Survey population

The survey was reviewed by the Institutional Review Board at the Johns Hopkins University School of Medicine and approved as a Quality Improvement project. Recipients of the survey were identified from two outpatient clinics in the Johns Hopkins Department of Psychiatry & Behavioral Sciences. The first, the Johns Hopkins Bayview Community Psychiatry Program (CPP), is a teaching hospital-based community mental health center of over 25 different ambulatory and school based clinical programs, including large adult and child outpatient programs. The second is the Johns Hopkins Resident Outpatient Continuity Clinic (ROCC), which is composed of psychiatry trainees and their clinical supervisors who provide both psychotherapy and pharmacological management to patients across the range of severe mental illnesses. Like the rest of the hospital system, these clinics made the abrupt transition to telepsychiatry in response to the pandemic between March 9, 2020 and March 16, 2020. Telepsychiatry remained the primary mode of service delivery for the next year including up to the deployment of the survey 1 year later as described below.

### The survey

The survey was developed based on a literature review of prior surveys on telemedicine in tandem with feedback from providers across the Johns Hopkins Department of Psychiatry and Behavioral Sciences. Multiple items were adapted for use in this survey from previously validated questionnaires, including the Telemedicine Satisfaction Scale (23); the Telemedicine Usability Questionnaire (24); the System Usability Scale (25); the mHealth App Usability Questionnaire (26); the Consumer Assessment of Healthcare Providers and Systems (CAHPS) Experience of Care and Health Outcomes Survey (ECHO) Version 3.0, including the Managed Behavioral Health Organization and the Managed Care Organization Supplemental Surveys (27, 28); the University of Washington Telemedicine Patient Satisfaction Survey (29); and an adapted CAHPS survey designed by Donelan et al. (9).

The final survey included up to four sections. The first section captured information on participant characteristics, including socio-demographics and other contextual features (i.e., different aspects of either telepsychiatry or in-person encounters, such as internet stability, privacy, or commute times). The second section asked participants about their experiences with telepsychiatry. Responses in this section were collected using a 5-item Likert scale, ranging from *not at all* to *very much.* The third section asked participants about their preferences between telepsychiatry and in-person care for specific facets of mental health care. Participants were able to select one of four options: *no opinion*, *no difference*, *in-person*, or *telemedicine*. The final section asked participants to indicate how the COVID-19 pandemic had affected different aspects of their mental health over the previous 3 months. Participants were asked about changes in different mental health symptoms and substance use during the COVID-19 pandemic and could select one of four response options: *never a problem*, *no change*, *decreased*, or *increased*.

Two different versions of the survey were constructed to target patients (Supplementary Table S1) and providers (Supplementary Table S2). The provider survey asked about provider specific experiences and preferences for telepsychiatry, but it did not include the fourth section on changes in mental health during the COVID-19 pandemic. The patient survey asked about patient specific experiences and preferences for telepsychiatry and included all four sections. For patients younger than 18, questions regarding substance use were removed from the fourth section.

### Survey dissemination

All patients who had an encounter in one of the two targeted clinics between January 1st, 2020 and February 4th, 2021 (for adult patients 18 years or older) or March 1st, 2021 (for child patients younger than age 18) were eligible to receive the survey. Providers practicing in these two clinics at the time were also eligible. All surveys were built in Qualtrics and disseminated by email. Provider surveys were disseminated on February 2nd, 2021. The patient surveys were disseminated to adult patients on February 16th and 17th, 2021 in the CPP and ROCC clinics, respectively, while they were distributed to child patients on March 4th, 2021 in CPP only. In the instance that the parent of a child received the survey, language was included asking the parent to share the survey with their child. Non-respondents received two follow-up emails per week for 8 weeks. The timing of these reminders fluctuated in an attempt to catch different patterns of availability. On March 31st and April 1st, 2021 300 adult non-responders were randomly selected for a telephone follow-up. Designated patients were called by one of two study team members and were given a reminder to complete the study, either on their own or via phone with the study team member if they preferred. Response collection was closed on May 14th, 2021.

### Statistical analysis

Patient and provider characteristics, their experiences with telepsychiatry, and their preferences for telepsychiatry vs. in-person care for different facets of care were reported separately as percentages of total respondents for that specific item. For the presentation of results for experiences with telepsychiatry, Likert responses were grouped into three levels: “*not at all* or *a little*”, *somewhat*, and “*moderately* or *very much*”. Separate logistic regression models were then used to examine the relationship between patient and provider characteristics and their experiences with telepsychiatry and their preferences for telepsychiatry vs. in-person care. For the examination of experiences with telepsychiatry, the primary analysis focused on overall satisfaction, while for preferences the primary analysis focused on preference for telepsychiatry vs. in-person care post-pandemic (i.e., “if the pandemic goes away”). In these models, the Likert responses for telepsychiatry experiences were dichotomized across the median response (lower than the median response vs. median or higher response). In instances where the median response was an extreme value (*not at all* or *very much*) responses were dichotomized as the median vs. all other values. Similarly, responses for treatment modality preferences (*telemedicine*, *in-person*, or *no difference*) were dichotomized as preference for “*telemedicine* or *no difference*” vs. *in-person* treatment (i.e., standard of care). All logistic regression models except those reported for exploratory analyses were adjusted for age, sex, and race. Results at *p* ≤ 0.05 were considered statistically significant. We did not correct for multiple testing because the *a priori* goal was to generate hypotheses about the range of features that might be associated with patient and provider experiences with and preferences for telepsychiatry. R version 4.1.1 was used for all statistical analysis.

## Results

### Patient characteristics

A total of 604 patients out of 3,017 (20%) completed the survey. Patient characteristics are listed in Table 1. The majority of patients were female (63%, *n* = 344) and White (74%, *n* = 383). Most patients had some education above the high school level (76%, *n* = 393). The majority of patients used either their computer (46%, *n* = 201) or video on their phone/tablet (42%, *n* = 182) for telepsychiatry visits and had reliable internet *all of the time* (76%, *n* = 392). A majority of patients were local, living within 30 min of their respective clinic (63%, *n* = 276).

**Table 1:** Characteristics of patient respondents.

	Patients *N* (%)
**Socio-demographics** ^a^	
*Sex*	
Female	344 (63%)
Male	201 (37%)
*Race*	
White	383 (74%)
Black	88 (17%)
Asian/PI	28 (5%)
Multiracial	13 (3%)
Native American	6 (1%)
*Age*	
31 or younger	123 (23%)
32–51	177 (33%)
52 or older	242 (45%)
*Education*	
High school or less	123 (24%)
Some college	111 (22%)
Associate’s/bachelor’s	140 (27%)
Postgraduate	142 (28%)
**Contextual features**	
*Device used for telehealth*	
Computer	201 (46%)
Phone/tablet (video)	182 (42%)
Phone (no video)	48 (11%)
Other	2 (0%)
*Internet access*	
Less than all of the time	127 (24%)
All of the time	392 (76%)
*Travel time to clinic (min)*	
0–30	276 (63%)
31–60	123 (28%)
61 or more	40 (9%)

a Percentages calculated based on the number of received responses for each specific item. Patients who did not respond to an item (i.e., missing) were not included in that item’s total.

### Provider characteristics

A total of 154 providers out of 250 (62%) completed the survey. Provider characteristics are listed in Table 2. The majority of providers were female (78%, *n* = 113) and White (78%, *n* = 111). Responding providers included mental health therapists (58%, *n* = 84), psychiatrists/physicians (24%, *n* = 35), and second to fourth post-graduate year (PGY-2–PGY-4) psychiatry resident physicians (16%, *n* = 21). Most providers used computers (76%, *n* = 102) for their telepsychiatry encounters and had internet access *all of the time* (77%, *n* = 111).

**Table 2:** Characteristics of provider respondents.

	Providers *N* (%)
**Socio-demographics** ^a^	
*Sex*	
Female	113 (78%)
Male	32 (22%)
*Race*	
White	111 (78%)
Black	17 (12%)
Asian/PI	9 (6%)
Multiracial	5 (4%)
Native American	0 (0%)
*Age*	
31 or younger	41 (30%)
32–51	63 (47%)
52 or older	31 (23%)
*Degree status*	
Therapist (PhD, PsyD, LCSW, LMSW)	84 (58%)
MD	35 (24%)
Other	25 (17%)
*Years in practice*	
Training (residency)	21 (16%)
Least experience (0.5–3 years)	26 (21%)
Below median experience (4–10 years)	26 (21%)
Above median experience (11–21 years)	24 (19%)
Most experience (22^+^ years)	29 (23%)
**Contextual features**	
*Device used for telehealth*	
Computer	102 (76%)
Phone/tablet (video)	20 (15%)
Phone (no video)	12 (9%)
Other	0 (0%)
*Internet access*	
Less than all of the time	35 (23%)
All of the time	111 (77%)
*Appointment location*	
Home office/other	83 (61%)
Work office	50 (36%)
Other	4 (3%)

a Percentages calculated based on the number of received responses for each specific item. Providers who did not respond to an item (i.e., missing) were not included in that item’s total.

### Patient experiences with telepsychiatry

**Figure 1:**
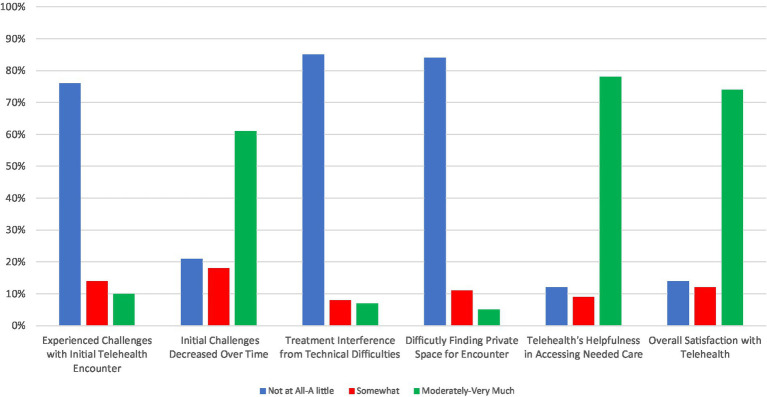
Patient experiences with telehealth rated on a Likert scale with response groups collapsed as “*not at all* or *a little*,” “*somewhat*,” and “*moderately* or *very much*.”

Summary results of patient experiences with telepsychiatry are shown in Figure 1 with more details provided in Supplementary Table S3. Most patients experienced few challenges (i.e., “*not at all* or *a little*”) with their initial telepsychiatry encounter (76%, *n* = 364). Further, most patients felt challenges with telepsychiatry “*moderately* or *very much*” decreased over time (61%, *n* = 147). The majority of patients said that technical difficulties interfered with treatment “*not at all* or *a little*” (85%, *n* = 405). Additionally, most patients responded that it was “*not at all* or *a little*” difficult to find private space for an encounter (84%, *n* = 395). Overall, there was a high level of satisfaction with telepsychiatry. Most patients (74%, *n* = 354) said they were “*moderately* or *very much*” satisfied using telepsychiatry to receive treatment, and most said it was “*moderately* or *very much*” helpful for accessing care during the pandemic (78%, *n* = 393).

Several features were associated with patient satisfaction in telepsychiatry (Table 3). The only socio-demographic feature was education; patients who had a post-graduate education were the most likely to be satisfied with telepsychiatry (OR = 2.13, 95% CI: 1.19–3.84). Technology and privacy-related features were also associated. Satisfaction was lower among patients who had poorer internet access (OR = 0.46, 95% CI: 0.28–0.73), initial set-up challenges (OR = 0.37, 95% CI: 0.2–0.55), technical difficulties that interrupted treatment (OR = 0.20, 95% CI: 0.13–0.30), or difficulty in finding private space to meet (OR = 0.36, 95% CI: 0.24–0.55). Conversely, patients were more likely to be satisfied with telepsychiatry if their initial set up challenges decreased over time (OR = 2.86, 95% CI: 1.57–5.33). Clinically, patients who experienced worsening mental health symptoms and substance use problems over the course of the pandemic were also less likely to be satisfied with telepsychiatry. This was particularly true for patients who reported worsening depression (OR = 0.38, 95% CI: 0.18–0.75), anxiety (OR = 0.41, 95% CI: 0.18–0.84), and suicidality (OR = 0.36, 95% CI: 0.13–0.94).

**Table 3:** Patient features and associations with satisfaction and preferences for telehealth.

	Satisfaction with telehealth OR (95% CI)^a^	Preference for telehealth post-pandemic OR (95% CI)^a^
**Socio-demographics**		
*Age (ref: ≤25 years)*		
>26 years	1.45 (0.81–2.58)	1.19 (0.65–2.19)
*Sex (ref: female)*		
Male	1.05 (0.70–1.57)	0.94 (0.62–1.43)
*Race (ref: White)*		
Black	0.87 (0.50–1.52)	1.45 (0.81–2.67)
Asian/PI	0.90 (0.39–2.13)	1.67 (0.70–4.26)
Multiracial	0.63 (0.18–2.16)	1.02 (0.27–4.22)
Native American	0.45 (0.06–2.76)	3.23 (0.47–63.92)
*Education (ref: HS or less)*		
Some college	1.16 (0.65–2.08)	1.62 (0.87–3.02)
AA/BA	1.47 (0.83–2.61)	**2.28 (1.24–4.26)**
Post-graduate	**2.13 (1.19–3.84)**	**1.96 (1.07–3.61)**
**Contextual features**		
*Device used (ref: computer)* ^b^		
Phone/tablet (video)	0.94 (0.60–1.46)	0.91 (0.57–1.45)
Phone (no video)	0.88 (0.45–1.77)	0.74 (0.37–1.49)
*Internet access (ref: all the time)*		
Less than all the time	**0.46 (0.28–0.73)**	**0.50 (0.31–0.82)**
*Travel time to clinic (ref: 0–30 min)*		
31–60 min	1.07 (0.66–1.73)	**1.72 (1.04–2.85)**
≥61 min	1.88 (0.86–4.41)	**2.35 (1.06–5.68)**
**Telehealth experiences** ^c^		
Initial setup challenge	**0.37 (0.25–0.55)**	0.52 (0.34–0.78)
Challenges decreased over time	**2.86 (1.57–5.33)**	**2.35 (1.27–4.44)**
Technical difficulties	**0.20 (0.13–0.30)**	**0.41 (0.26–0.63)**
Difficulty finding private space	**0.36 (0.24–0.55)**	0.91 (0.60–1.39)
Helpful in access care	**45.70 (24.33–92.87)**	**8.43 (5.20–13.99)**
**Mental health changes** ^d^		
Depression	**0.38 (0.18–0.75)**	**0.36 (0.16–0.76)**
Anxiety	**0.41 (0.18–0.84)**	**0.40 (0.17–0.85)**
Anger	0.56 (0.26–1.16)	**0.41 (0.17–0.91)**
Suicidality	**0.36 (0.13–0.94)**	0.63 (0.23–1.65)
Difficulty sleeping	0.70 (0.32–1.47)	0.64 (0.28–1.42)
Difficulty concentrating	0.47 (0.21–0.99)	0.54 (0.24–1.15)
Alcohol use	0.61 (0.22–1.62)	0.82 (0.27–2.41)
Tobacco use	0.75 (0.17–3.21)	0.30 (0.61–1.30)
Marijuana use	0.38 (0.08–1.67)	0.60 (0.11–3.26)

OR, Odds Ratio; CI, confidence intervals; ref, reference; PI, Pacific Islander; HS, high school; AA, associate of arts; Ba, bachelor of arts.

a Logistic regression models were estimated separately for each patient-related feature and the two different dependent variables (satisfaction with telehealth and patient preference for telehealth post-pandemic) controlling for age, sex and race. Satisfaction with telehealth was dichotomized at the median response which was *very much* vs. all other responses. Patient preference for telehealth post-pandemic compared responses in favor of telehealth and telehealth and in-person equivalently vs. in-person care. Bolded results are significant at *p* < 0.05.

b The “other” category of devices used for telehealth was not included in the models due to small sample size.

c Independent variables for each telehealth experience were dichotomized at the median response (see Supplementary Table S3 for details). The reference group in the analysis for each variable is the lower than median response. For instances when the median was an extreme response (i.e., “*not at all*” or “*very much*,” the bin containing the lower values was the reference group).

d Independent variables for changes in mental health status during the pandemic compared those whose symptoms worsened vs. improved. Patients who reported no change during the pandemic were excluded. The reference group in the analysis for each variable is the “*symptoms improving*” response.

### Patient preferences for telepsychiatry

**Figure 2:**
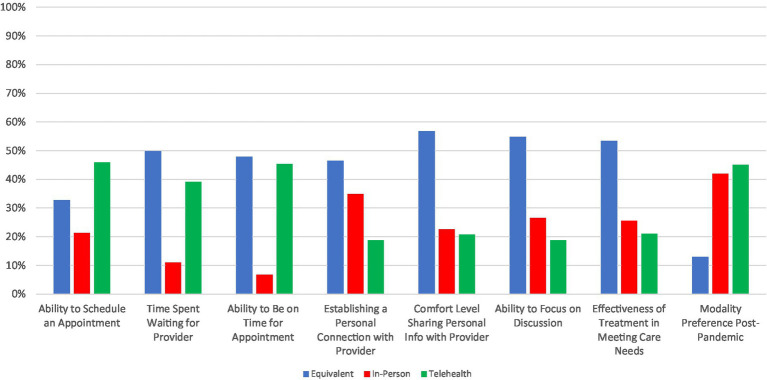
Patient preferences of treatment modality for different care related features.

Summary results of patient responses for whether they prefer telepsychiatry, in-person treatment, or view both modalities as equivalent are shown in Figure 2, with more details provided in Supplementary Table S4. Patients more frequently rated telepsychiatry and in-person care as equivalent for many of the facets queried, including amount of time waiting for an appointment to start (50%), ability to be on time for an appointment (48%), establishing a personal connection with the provider (46%), comfort level sharing personal information with the provider (57%), ability to focus on the discussion (55%), and effectiveness of treatment to meet patient needs (53%). Preference for telepsychiatry was endorsed most frequently when considering the ability to schedule a time to meet with a provider (46%), while in-person care was not rated most often for any of the facets probed. There was a relatively even split in preference for telepsychiatry vs. in-person care when anticipating treatment post-pandemic: 45% reported preferring telepsychiatry compared with 42% for in-person care, while only a small minority of 13% rated them equivalently.

Many of the same features that were associated with patients’ overall satisfaction with telepsychiatry were also associated with their preference to continue with it post-pandemic (Table 3). Education was again relevant as patients with an Associates/Bachelor’s degree (OR = 2.28, 95% CI: 1.24–4.26) or post-graduate degree (OR = 1.96, 95% CI: 1.07–3.61) were more likely to prefer telepsychiatry in a post-pandemic setting, compared to those with a high school education or less. Patients with longer travel times to appointments were also more likely to prefer telepsychiatry post-pandemic. Here, there was a clear duration effect: compared to those who travel 0–30 min to the clinic, those who travel 31–60 min were more likely to prefer telepsychiatry (OR = 1.72, 95% CI: 1.04–2.85), and this preference was even stronger for those who travel over an hour (OR = 2.35, 95% CI: 1.06–5.68). Patients with poorer internet access were less likely to prefer telepsychiatry post-pandemic (OR = 0.50, 95% CI: 0.31–0.82). Experiences with telepsychiatry and changes in mental health during the pandemic were again also associated with preferences for seeking care post-pandemic. Patients who experienced technical difficulties with telepsychiatry were less likely to endorse a preference for telepsychiatry (OR = 0.41, 95% CI: 0.26–0.63), while patients who found that the technical challenges decreased over time (OR = 2.35, 95% CI: 1.27–4.44) and telepsychiatry was useful for accessing care (OR = 8.43, 95% CI: 5.20–13.99) were more likely to prefer telepsychiatry for future care. Clinically, patients who experienced worsening symptoms of depression (OR = 0.36, 95% CI: 0.16–0.76), anxiety (OR = 0.40, 95% CI: 0.17–0.85) and anger (OR = 0.41, 95% CI: 0.17–0.91) were less likely to prefer telepsychiatry post-pandemic. Finally, as with overall satisfaction, we again observed that age, sex and race were not associated with patient preferences for telepsychiatry vs. in-person care. However, in an exploratory analysis of patient preferences, we observed that Black patients found it easier to establish a personal connection via telepsychiatry (OR 2.3, 95% CI: 1.23–4.58) and easier to focus on the discussion (OR 2.45, 95% CI: 1.22–5.49) compared to White patients.

### Provider experiences with telepsychiatry

**Figure 3:**
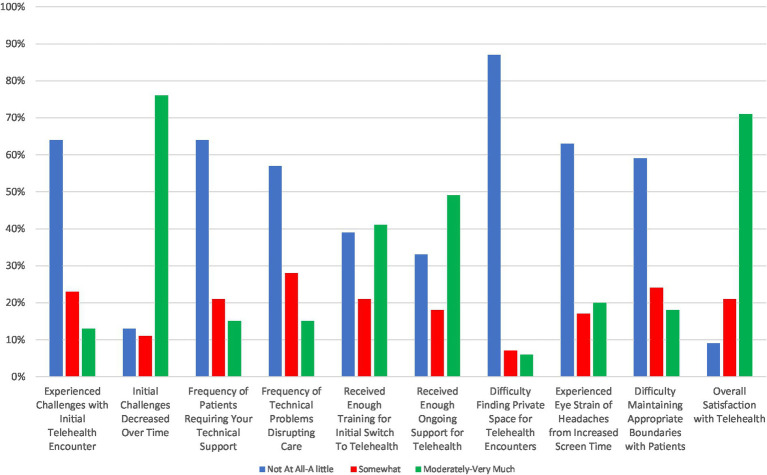
Provider experiences with telehealth rated on a Likert scale with response groups collapsed as “*not at all or a little*,” “*somewhat*,” and “*moderately or very much*.”

Provider experiences with telepsychiatry are shown in Figure 3, with more details provided in Supplementary Table S5. Most said they had few (i.e., “*not at all* or *a little*”) challenges (64%, *n* = 85) with initial encounters and that the challenges decreased “*moderately* or *very much*” over time (76%, *n* = 77). Moreover, most providers reported they had few (i.e., “*not at all* or *a little*”) difficulties with finding private space for telepsychiatry encounters (87%, *n* = 116), experiencing eye strain or headache from increased screen time (63%, *n* = 82), maintaining appropriate boundaries with patients (59%, *n* = 77), providing tech support to patients (64%, *n* = 84), or resolving technical problems that disrupted care (57%, *n* = 86). A notable minority of providers reported they received insufficient (i.e., “*not at all* or *a little*”) training for the initial telepsychiatry encounter (39%, *n* = 51) and on-going technical support (33%, *n* = 44). However, overall satisfaction with telepsychiatry was high with 71% (*n* = 93) of providers reporting they were “*moderately or very much*” satisfied with telepsychiatry.

Providers were more likely to be satisfied with telepsychiatry if they felt they had received sufficient initial training (OR = 2.70, 95% CI: 1.14–6.56) and on-going support (OR = 2.51, 95% CI: 1.02–6.29) or if the initial challenges with telepsychiatry decreased over time (OR = 4.36, 95% CI: 1.66–12.20) (Table 4). On the other hand, providers who experienced on-going technical challenges with telepsychiatry encounters (OR = 0.12, 95% CI: 0.01–0.64) and who experienced eye strain as a result of these encounters (OR = 0.42, 95% CI: 0.15–1.11) were less likely to be satisfied. In addition, providers who joined appointments from the office instead of home were also less satisfied with telepsychiatry (OR = 0.21, 95% CI: 0.08–0.50). No socio-demographic factors were significantly associated with provider satisfaction with telepsychiatry. There was a trend towards more experienced clinicians being less likely to be satisfied with telepsychiatry, but the differences across provider training levels were not significant.

**Table 4:** Provider features and associations with satisfaction and preferences for telehealth.

Provider features	Satisfaction with telehealth OR (95% CI)^a^	Preference for telehealth post-pandemic OR (95% CI)^a^
**Socio-demographics**		
*Age (ref: ≤31 years)*		
32–51 years	1.16 (0.44–3.03)	0.45 (0.18–1.05)
>51 years	0.86 (0.27–2.75)	0.61 (0.20–1.86)
*Sex (ref: female)*		
Male	0.83 (0.32–2.25)	0.99 (0.40–2.45)
*Race (ref: White)* ^b^		
Black	0.58 (0.15–2.47)	0.75 (0.19–3.00)
Asian/PI	2.39 (0.37–47.07)	0.99 (0.24–4.39)
Multiracial	0.78 (0.07–18.12)	0.47 (0.02–5.34)
*Degree (ref: therapist)*		
MD	0.76 (0.27–2.21)	2.07 (0.78–5.82)
Other	1.32 (0.34–6.72)	2.16 (0.62–8.22)
*Clinical experience (ref: least exp)*		
Resident	0.45 (0.09–1.96)	0.96 (0.25–3.71)
Below median exp	0.82 (0.16–4.35)	0.51 (0.14–1.84)
Above median exp	0.53 (0.08–3.09)	0.37 (0.08–1.66)
Most exp	0.36 (0.05–2.57)	0.37 (0.06–1.95)
**Contextual features**		
*Device used (ref: computer)*		
Phone/tablet (video)	1.19 (0.34–4.90)	0.38 (0.11–1.24)
Phone (no video)	0.77 (0.21–3.26)	1.82 (0.50–7.70)
*Internet access (ref: all the time)*		
Less than all the time	0.69 (0.28–1.77)	**0.35 (0.13–0.86)**
*Appointment location (ref: home)*		
Work office	**0.21 (0.08–0.50)**	**0.41 (0.17–0.96)**
Other	0.33 (0.03–7.51)	1.36 (0.11–31.62)
**Telehealth experiences** ^c^		
Initial setup challenge	0.50 (0.15–1.44)	0.43 (0.16–1.11)
Challenges decreased over time	**4.36 (1.66–12.20)**	1.75 (0.71–4.40)
Technical difficulties	**0.12 (0.01–0.64)**	**0.13 (0.02–0.46)**
Difficulty finding private space	0.81 (0.33–1.99)	0.59 (0.25–1.37)
Sufficient initial training	**2.70 (1.14–6.56)**	1.56 (0.68–3.62)
Sufficient on-going support	**2.51 (1.02–6.29)**	1.70 (0.72–4.13)
Experience eye strain	**0.42 (0.15–1.11)**	0.48 (0.20–1.13)
Maintain appropriate boundaries	0.81 (0.25–2.31)	**0.28 (0.09–0.81)**

OR, odds ratio; CI, confidence intervals; ref, reference; PI, Pacific Islander; exp, experience.

a Logistic regression models were estimated separately for each provider-related feature and the two different dependent variables (satisfaction with telehealth and provider preference for telehealth post-pandemic) controlling for age, sex and race. Satisfaction with telehealth was dichotomized at the median response which was *moderately* and *very much* vs. all other responses. Provider preference for telehealth post-pandemic compared responses in favor of telehealth and telehealth and in-person equivalently vs. in-person care. Bolded results are significant at *p* < 0.05.

b A separate logistic regression model employing Black providers as the reference group demonstrated that there were no statistically significant differences in satisfaction or preference between Black and Asian providers.

c Independent variables for each telehealth experience were dichotomized at the median response (see Supplementary Table S5 for details). The reference group in the analysis for each variable is the lower than median response. For instances when the median was an extreme response (i.e., “*not at all*” or “*very much*,” the bin containing the lower values was the reference group).

### Provider preferences for telepsychiatry

**Figure 4:**
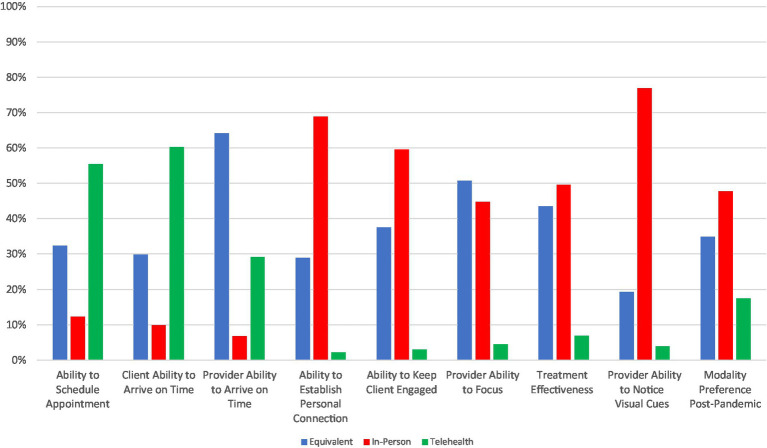
Provider preferences of treatment modality for different care related features.

Summary results of provider preferences for telepsychiatry vs. in-person treatment modalities are shown in Figure 4, with more details provided in Supplementary Table S6. Similar to patients, providers tended to prefer telepsychiatry for administrative-related purposes. A majority of providers preferred telepsychiatry for the ability to schedule appointments (55%) and for facilitating patient punctuality (60%). However, providers rated telepsychiatry and in-person care as equivalent (64%) when considering their own punctuality. By contrast, providers notably preferred in-person care for therapeutic alliance aspects, including the ability to notice visual cues (77%), keep the patient engaged (60%), and establish a personal connection with the patient (69%), as well as for overall treatment effectiveness (50%). Perhaps as a result, and in contrast with patients, a more distinct plurality of providers preferred in-person (48%) vs. telepsychiatry (17%) care post-pandemic, with 35% rating them equivalently.

Providers were less likely to prefer telepsychiatry as a post-pandemic modality if they experienced worse internet access (OR = 0.35, 95% CI: 0.13–0.86), technical difficulties (OR = 0.13, 95% CI: 0.02–0.46), or challenges maintaining appropriate boundaries with clients (OR = 0.28, 95% CI: 0.09–0.81) (Table 4). In addition, providers who did most of their telepsychiatry encounters from their work office also were less likely to prefer telepsychiatry for post-pandemic encounters (OR = 0.41, 95% CI: 0.17–0.96). Similar to what was observed for overall satisfaction, provider socio-demographics and level of training were not associated with their modality preference post-pandemic. However, in exploratory analyses to examine these relationships in more detail, we noted that Black providers were less likely than White providers to feel like they had been provided enough ongoing support with the transition to telepsychiatry (OR 0.27, 95% CI: 0.07–0.97). This did not translate into their being more or less likely to be satisfied with telepsychiatry or prefer it in the future.

## Discussion

This paper examined patient and provider experiences and preferences in using telepsychiatry during the COVID-19 pandemic. Overall, patients and providers reported that telepsychiatry was acceptable and worked well, with over 70% of patients and providers reporting they were satisfied with telepsychiatry for delivering mental health care. However, there were greater divergences between patients and providers when asked whether they would prefer telepsychiatry or in-person care after the pandemic. Patients were more evenly divided with 45% preferring telepsychiatry compared to 42% for in-person care, and only 13% rating them equivalently. By contrast, providers were more likely to favor in-person care over telepsychiatry by 48% to 17%, with 35% rating them equivalently.

The high level of overall satisfaction that we observed is consistent with a large body of previous work carried out both before and during the pandemic (3, 9–13, 16, 18–20). For example, both Gentry et al. (6) and Steidtmann et al. (21) recently reported finding high levels of satisfaction with telepsychiatry among providers and clinical staff with patient contact during the pandemic. Both studies also reported high levels of patient satisfaction, which was collected indirectly through provider impression (6, 21).

It is less clear if the observed overall satisfaction with telepsychiatry translates into a preference among patients and providers to continue to use telepsychiatry after the pandemic. In a survey at the University of Michigan Outpatient Psychiatry Clinics, a little over half of the patients (54.1%) responded they would likely continue telepsychiatry even if in-person appointments were resumed (30). This echos our finding that 55% of patients rated telepsychiatry and in-person care equivalently or would prefer telepsychiatry if the pandemic abated. Another survey of clinicians at Mass General Hospital Outpatient Psychiatry Clinics reported a strong preference to continue telepsychiatry after the pandemic, although percentages were not given (20). Similarly, both Gentry et al. (6) and Steidtmann et al. (21) reported a strong preference on the part of the provider to continue telepsychiatry after the pandemic, at least for a significant portion of their caseload (6, 21). By contrast, Hunsinger et al. (31) found that only 17% of providers and 25% of patients preferred telepsychiatry. The lower rates reported by providers in this study were similar to what we observed in our survey.

Several factors were associated with overall satisfaction and preference for telepsychiatry among both patients and providers. Technical considerations such as access to stable internet were the most consistently reported factors affecting both satisfaction and future modality preference. Both patients and providers with less than fully stable internet access had more challenging telepsychiatry experiences overall and were significantly less likely to be satisfied with it and to want to continue using it in the future. This makes intuitive sense and is consistent with previous studies. For example, a pre-pandemic cross-sectional study of patients in the Kaiser system in California found a higher likelihood of telemedicine use in neighborhoods with high rates of residential internet access (32). In addition to stable internet access, other technical challenges experienced by patients and providers in using telemedicine had a similarly strong impact on their attitudes towards it. These findings highlight the importance of a robust internet infrastructure to sustain widespread use of telemedicine and technical support to overcome challenges when using audio/video technology.

Education was also strongly associated with overall satisfaction and preference for telepsychiatry among patients. Patients with higher levels of education were more likely to be satisfied with telepsychiatry and prefer using it post-pandemic. One possible explanation is that patients with higher education were also less likely to experience initial technical challenges with telemedicine and more likely to experience a decrease in those challenges over time (data not shown). As noted previously, technical issues significantly impact overall satisfaction and preferences. Education and overall clinical experience were less clearly associated with satisfaction and preferences for telemedicine among providers. There was a trend suggesting that providers with more experience, especially above the median level, were less satisfied with and less likely to prefer telemedicine, but these trends were not significant.

Other sociodemographic factors including age, sex and race were not associated with overall satisfaction or preference for telepsychiatry for both patients and providers. With regard to race, several previous studies have reported greater use and preference for telemedicine among Black patients. For example, a large pre-COVID-19 study from Kaiser on telemedicine use in primary care found that Black patients were significantly more likely than White patients to choose telemedicine over in-person care (32). In addition, the Pew Research Center’s American Trends Panel study found that of all racial/ethnic groups, Black patients were most likely to use telemedicine during the pandemic (33). However, not all studies have found significant associations between race and telemedicine preferences and experiences (30). Although we did not find significant associations either, we did observe several revealing differences in the telepsychiatry experiences of both patients and providers of color that warrant consideration.

We found that Black patients were over two times more likely to find it easier to establish a personal connection and focus on treatment sessions via telepsychiatry compared to White patients. It is unclear from our findings why this is the case, but it merits further investigation to better understand why certain patient populations differ in their experience with telepsychiatry in ways that may affect their care. We also noted that Black providers were less likely than White providers to feel like they had received enough support when switching to telepsychiatry. Although this did not translate into differences in overall satisfaction or preference for telepsychiatry, it demands greater attention in the future. The transition to telepsychiatry was swift with minimal opportunities for formalized training. Such training will likely be important to increase acceptance and satisfaction with telepsychiatry going forward. Previous studies have shown that appropriate training in different areas of telemedicine (such as “digital psychiatry”) strongly influence providers’ perceptions and attitudes regarding the usefulness and effectiveness of remote care (34–36). Indeed, we observed that all providers who felt they received sufficient initial training and on-going support were more than two times as likely to be satisfied with telepsychiatry. The structural factors leading to Black providers feeling less supported at work will need to be addressed to minimize potential disparities in using telepsychiatry.

Finally, we observed that the mental health status of patients strongly influences their attitudes towards telepsychiatry. While there has been some discussion about which mental illnesses are better suited for care via telemedicine, there has been more limited investigation of how the severity or progression of these illnesses is associated with patient attitudes towards telemedicine (37). One small survey from Louisiana State University Health Sciences Center did not find an association between self-report of severity of anxiety/depression symptoms and visit type preference (31). We found that worsening mental health and substance use problems, particularly depression and anxiety, were significantly associated with lower satisfaction and preference for telepsychiatry. Patients with worsening mental health and substance use problems may have been frustrated at the perceived lack of progress in their treatment, and thus less happy with treatment overall. As providers move towards a hybrid model, patients who are not improving may benefit from the option to return to in-person care. Thus, in the future, it may be important for providers to carefully monitor their patient’s clinical status when making decisions about the optimal modality of treatment.

### Limitations

Our study had several limitations. The response rates were 20% for patients and 62% for providers, which leaves open the possibility of response bias. However, several steps were taken to minimize this risk. Email reminders to non-responders were distributed twice per week on different days and times, in an attempt to collect responses from patients on different schedules. Furthermore, a telephone follow-up (see Methods) of 300 randomly selected non-responders was employed to minimize bias due to convenience sampling as well as to recruit patients who do not have uniform access to email. However, future work that evaluates the efficacy of telepsychiatry should work to employ a more sustained multi-modal collection effort (e.g., physical mailers and a broader telephone follow-up) to both improve the response rate and to minimize concerns of exacerbating the digital divide (4, 5). In addition, despite having a more diverse sample compared to similar studies, our respondents were still relatively homogeneous with the majority identifying as White and female. Follow-up studies should work to ensure that their samples better reflect the socio-demographic makeup of the participating communities or clinics through more targeted recruitment efforts. Lastly, we did not collect data on the encounter level or at the level of individual patient-provider relationships. As noted by Sabin and Skimming (2) the acceptability of telepsychiatry can change across therapeutic relationships, specific diagnoses (including severity), or even the type of service (e.g., support for caregivers) (2, 5). Further research should seek to investigate how satisfaction with telepsychiatry can change across different patient-provider dyads as well as over time.

### Implications and conclusion

Due to the COVID-19 pandemic, mental health clinics were forced to quickly adopt telemedicine to continue delivering care to patients with mental health and substance use problems. The rapid nature of the transition to telepsychiatry necessitates a re-evaluation of how patients and providers perceive utilization of remote care, in order to reveal the benefits and limitations of using telemedicine in psychiatry. Now that the emergency pandemic measures have receded, it is an open question to what extent telepsychiatry will continue to be used to deliver care (15). We found that both patients and providers were generally satisfied with telepsychiatry, especially as a means to continue accessing/providing care during the pandemic when in-person access to care was not feasible. However, there were greater differences in preferences for continuing to use telepsychiatry after the pandemic. The findings reported here can help to inform what may promote positive patient and provider experiences with telepsychiatry, as well as what areas or aspects may require additional resources in order to ensure a more just and equitable practice of telepsychiatry.

## Data availability statement

The raw data supporting the conclusions of this article will be made available by the authors, without undue reservation.

## Author contributions

MM, IC, MW, AB, LM, WN, BS, JS, PZ, and AR contributed to the study conception and design and commented on previous versions of the manuscript. MM, IC, AB, JS, PZ, and AR were responsible for data collection and subsequent analysis. The first draft of this manuscript was written by MM, IC, PZ, and AR. All authors contributed to the article and approved the submitted version.

## Abbreviations

CPP: Community Psychiatry Program
ROCC: Resident Outpatient Continuity Clinic
CAHPS: Consumer Assessment of Healthcare Providers and Systems
ECHO: Experience of Care and Health Outcomes Survey
PGY: Post-Graduate Year (Residency Status)
OR: Odds Ratio
CI: Confidence Interval
