# Supplementary material for: Determinants of experience & satisfaction in telehealth psychiatry during the COVID-19 pandemic for patients & providers
Source: Front Psychiatry. 2023 Sep 1;14:1237249. doi: 10.3389/fpsyt.2023.1237249 (PMC10502508; doi:10.3389/fpsyt.2023.1237249)
Supplement: Supplementary file 1 [file Table_1.docx]

**Supplemental Table 1. Patient Survey**

| **Basic Information** |
| --- |
| Age |
| Sex |
| Race |
| Ethnicity |
| Highest level of education |
| What is the average travel time in minutes for you to get to an in-person appointment with your mental health provider? |
| Do you currently have reliable access to internet at your home? |
| Do you currently have a smartphone? |
| **Impact of COVID-19 on Mental Health** |
| Feeling down, depressed, or hopeless |
| Feeling nervous, anxious or on edge |
| Feeling angry or irritable |
| Thinking about suicide |
| Difficulty falling or staying asleep |
| *Difficulty concentrating on your regular activities |
| *Drinking alcoholic beverages |
| *Smoking cigarettes/e-cigarettes or using other tobacco products |
| *Smoking marijuana or using other marijuana products |
| *Using other illegal drugs |
| **Impact of COVID-19 on Mental Health Care Services** |
| Has the COVID-19 pandemic made it more difficult for you to get the mental health care you want? |
| Have you ever had an in-person appointment with your mental health provider? |
| Since the start of the COVID-19 pandemic, have you had a telehealth appointment with your mental health provider? |
| ^1^*If yes*: About how many telehealth appointments have you had with your mental health provider? |
| *^1^If yes*: What device do you use most of the time for telehealth appointments with your mental health provider? |
| ^1^*If yes*: Do most of your telehealth appointments include video or voice only? |
| *^1^If voice only*: What reason best describes why you are not using video? |
| ^1^*If no*: skip to the last question |
| **Attitudes Towards Using Telehealth** |
| Was it challenging learning how to set up and do a telehealth appointment for the first time? |
| ^1^Have the challenges with doing telehealth appointments decreased over time? |
| Do technical difficulties with the telehealth appointments interfere with your treatment? |
| Is it difficult to find a private space to use during a telehealth appointment? |
| Overall are you satisfied with using telehealth to get treatment? |
| Have the telehealth appointments been helpful to get the care you wanted during the COVID-19 pandemic? |
| **Preferences for Telehealth Versus In-person** |
| Ability to schedule a time to meet with my mental health provider |
| Amount of time I spend waiting for my mental health provider |
| My ability to be on time for my appointment |
| Establishing a personal connection with my mental health provider |
| Comfort level sharing personal/private information with my mental health provider |
| Ability to focus on the discussion with my mental health provider |
| Effectiveness of treatment in meeting my needs |
| If the COVD-19 pandemic goes away, which method would you prefer for meeting with your mental health provider |
| If the COVID-19 pandemic goes away, would you like to have the option to do telehealth visits in the future? |

Survey prompts for adult and children (younger than 18 years old) patients.

*Not asked for children patients

^1^Implies branching logic based on the participant’s response to an earlier item

**Supplemental Table 2. Provider Survey**

| **Basic Information** |
| --- |
| Age |
| Sex |
| Race |
| Ethnicity |
| Professional Degree |
| Years Practicing (*If in a residency or fellowship program and are not yet practicing independently, please enter '0'*) |
| ^1^Please enter training status |
| Do you currently have reliable access to internet at your home? |
| **Impact of COVID-19 on Mental Health Care Services** |
| What best describes the age breakdown of the majority of your patients? |
| Have you, as the provider, had both in-person and telehealth appointments? |
| Where do you conduct the majority of your telehealth appointments? |
| What type of device do you use during the majority of your telehealth appointments? |
| What is your estimate of the proportion of your patients that you do voice only telehealth appointments with? |
| ^1^For the patients that you do voice only telehealth appointments, what are the reasons? |
| **Attitudes Towards Using Telehealth** |
| Was it challenging learning how to set up and do a telehealth appointment for the first time? |
| ^1^Have the challenges with doing telehealth appointments decreased over time? |
| How frequently do you need to help your patient troubleshoot technical difficulties? |
| How frequently are your telehealth appointments disrupted by technical issues? |
| Do you feel you got enough training to make the switch to doing telehealth appointments? |
| Do you feel you get enough on-going support for doing telehealth appointments? |
| Is it difficult to find a private space to use for a telehealth appointment? |
| Overall are you satisfied with using telehealth to give treatment? |
| Have you experienced eye strain or headaches associated with increased screen time due to your telehealth appointments? |
| Have you experienced difficulty with patients maintaining appropriate boundaries for the clinical relationship during the telehealth appointments (for example inappropriate clothing/undressed; inappropriate setting [patient is driving]) |
| **Preferences for Telehealth Versus In-person** |
| Ability to schedule a time to meet with my patient |
| Ability of my patients to make their appointment on time |
| My ability to be on time for my appointment |
| Establishing a personal connection with my patient |
| Ability to keep patient/client engaged in the session |
| Ability to focus on the discussion with my patient |
| Effectiveness of treatment to meet my patient's needs |
| Ability to pick up on visual cues about my patient's mental health status |
| If the COVID-19 pandemic continues, would you prefer a telehealth appointment or an in-person appointment with appropriate PPE? |
| If the COVID-19 pandemic goes away, which method would you prefer for meeting with your patients? |
| If the COVID-19 pandemic goes away, would you like to have the option to do telehealth visits in the future? |

Survey prompts for participating providers

^1^Implies branching logic based on the participant’s response to an earlier item

**Supplemental Table 3. Details on Patient Experiences with Telehealth**

| **Patient Experience Prompts** | **Not at All**  **N (%)** | **A Little**  **N (%)** | **Somewhat**  **N (%)** | **Moderately**  **N (%)** | **Very Much**  **N (%)** |
| --- | --- | --- | --- | --- | --- |
| **Experienced Challenges with Initial Telehealth Encounter** | 231 (48%) | 133 (28%)* | 65 (14%) | 23 (5%) | 26 (5%) |
| **Initial Challenges Decreased Over Time** | 25 (10%) | 28 (11%) | 44 (18%) | 31 (13%)* | 116 (48%) |
| **Treatment Interference from Technical Difficulties** | 300 (63%)* | 105 (22%) | 38 (8%) | 18 (4%) | 12 (3%) |
| **Difficulty Finding Private Space for Encounter** | 302 (64%)* | 93 (20%) | 54 (11%) | 16 (3%) | 10 (2%) |
| **Telehealth’s Helpfulness in Accessing Needed Care** | 26 (5%) | 35 (7%) | 43 (9%) | 66 (14%)* | 303 (64%) |
| **Overall Satisfaction with Telehealth** | 31 (7%) | 31 (7%) | 57 (12%) | 87 (18%)* | 267 (56%) |

Numerical breakdown of data presented in Figure 1. All percentages calculated from number of responses to specific query (NA or missing data not included).

* Represents category demarking the median split for the specific prompt

**Supplemental Table 4. Details on Patient Preferences for Telehealth vs In-Person**

| **Patient Preference Prompt** | **Equivalent**  N (%) | **In-person**  N (%) | **Telehealth**  N (%) |
| --- | --- | --- | --- |
| **Ability to Schedule an Appointment** | 145 (33%) | 94 (21%) | 203 (46%) |
| **Time Spent Waiting for Provider** | 200 (50%) | 44 (11%) | 157 (39%) |
| **Ability to be on Time for Appointment** | 212 (48%) | 30 (7%) | 201 (45%) |
| **Establishing a Personal Connection with Provider** | 208 (46%) | 156 (35%) | 84 (19%) |
| **Comfort Level Sharing Personal Info with Provider** | 257 (57%) | 102 (23%) | 94 (21%) |
| **Ability to Focus on the Discussion** | 246 (55%) | 119 (27%) | 84 (19%) |
| **Effectiveness of Treatment in Meeting Care Needs** | 234 (53%) | 112 (26%) | 92 (21%) |
| **Modality Preference Post-Pandemic** | 55 (13%) | 186 (42%) | 198 (45%) |

Numerical breakdown of data presented in Figure 2. All percentages calculated from number of responses to specific query (NA or missing data not included).

**Supplementary Table 5. Details on Provider Experiences with Telehealth**

| **Provider Experience Prompts** | **Not At All**  **N (%)** | **A Little**  **N (%)** | **Somewhat**  **N (%)** | **Moderately**  **N (%)** | **Very Much**  **N (%)** |
| --- | --- | --- | --- | --- | --- |
| **Experienced Challenges with Initial Telehealth Encounter** | 32 (24%) | 53 (40%)* | 31 (23%) | 13 (10%) | 4 (3%) |
| **Initial Challenges Decreased Over Time** | 1 (1%) | 12 (12%) | 11 (11%) | 18 (18%)* | 59 (58%) |
| **Frequency of Patients Requiring Your Technical Support** | 29 (22%) | 55 (42%)* | 28 (21%) | 19 (14%) | 1 (1%) |
| **Frequency of Technical Problems Disrupting Care** | 20 (15%) | 56 (42%)* | 37 (28%) | 17 (13%) | 3 (2%) |
| **Received Enough Training for Initial Switch To Telehealth** | 26 (20%) | 25 (19%) | 28 (21%)* | 32 (24%) | 22 (17%) |
| **Received Enough Ongoing Support for Telehealth** | 21 (16%) | 23 (17%) | 24 (18%)* | 34 (26%) | 31 (23%) |
| **Difficulty Finding Private Space for Telehealth Encounters** | 89 (67%)* | 27 (20%) | 9 (7%) | 5 (4%) | 3 (2%) |
| **Experienced Eye Strain of Headaches from Increased Screen Time** | 47 (36%) | 35 (27%)* | 22 (17%) | 15 (11%) | 12 (9%) |
| **Difficulty Maintaining Appropriate Boundaries with Patients** | 30 (23%) | 47 (36%)* | 31 (24%) | 14 (11%) | 9 (7%) |
| **Overall Satisfaction with Telehealth** | 2 (2%) | 9 (7%) | 28 (21%) | 43 (33%)* | 50 (38%) |

Numerical breakdown of data presented in Figure 3. All percentages calculated from number of responses to specific query (NA or missing data not included).

* Represents category demarking the median split for the specific prompt

**Supplementary Table 6. Details on Provider Preferences for Telehealth vs In-Person**

| **Provider Preference Prompts** | **Equivalent**  **N (%)** | **In-Person**  **N (%)** | **Telehealth**  **N (%)** |
| --- | --- | --- | --- |
| **Ability to Schedule Appointment** | 42 (32%) | 16 (12%) | 72 (55%) |
| **Ability of Client to Arrive on Time** | 39 (30%) | 13 (10%) | 79 (60%) |
| **Ability of Provider to Arrive on Time** | 86 (64%) | 9 (7%) | 39 (29%) |
| **Ability to Establish Personal Connection** | 39 (29%) | 93 (69%) | 3 (2%) |
| **Ability to Keep Client Engaged** | 51 (38%) | 81 (60%) | 4 (3%) |
| **Ability of Provider to Focus** | 68 (51%) | 60 (45%) | 6 (4%) |
| **Treatment Effectiveness** | 57 (44%) | 65 (50%) | 9 (7%) |
| **Ability of Provider to Notice Visual Cues** | 25 (19%) | 100 (77%) | 5 (4%) |
| **Modality Preference Post-Pandemic** | 46 (35%) | 63 (48%) | 23 (17%) |

Numerical breakdown of data displayed in Figure 4. All percentages calculated from number of responses to specific query (NA or missing data not included).
